# Preparation of Transparent and Scratch-Resistant Organic–Inorganic Hybrid Coatings: Role and Mechanism of Silane-Modified Nano-SiO_2_

**DOI:** 10.3390/polym18060674

**Published:** 2026-03-10

**Authors:** Shilu Wang, Siwei Hu, Hanhui Kang, Yongbin Li, Chunxiao Yin, Yuteng Ling, Haolan Xiao, Lili Wu

**Affiliations:** 1 Changda Municipal Engineering (Guangdong) Co., Ltd., No. 3, Heguang Street, Cuixiang New District, Zhongshan 528451, China; 67583555@163.com (S.W.); 13925148648@139.com (S.H.); li18825119721@163.com (Y.L.); lyt1612706676@163.com (Y.L.); 2 Guangdong Provincial Transportation Engineering Cost Affairs Center, No. 27 Baiyun Road, Yuexiu District, Guangzhou 510199, China; 18038867198@139.com; 3 School of Materials Science and Engineering, Wuhan University of Technology, Wuhan 430070, China; h_aron@petalmail.com

**Keywords:** scratch resistance, optical transmittance, silica sol, nano-silica, silane coupling agent

## Abstract

Optical plastics possess excellent optical and mechanical properties but are limited by poor surface hardness and scratch resistance. Herein, UV-curable organic–inorganic hybrid coatings were developed to enhance scratch resistance while maintaining high optical transparency. Nano-silica sols were prepared via tetraethoxysilane (TEOS) hydrolysis and surface modified with silane coupling agents (KH-560, KH-570, and KH-550) to improve their dispersion and interfacial reactivity in a polyurethane acrylate (PUA) matrix. The modified nano-silica was incorporated into a UV-curable PUA system to fabricate transparent composite coatings. The influences of nano-silica type and loading on hardness, flexibility, wettability, scratch resistance, and UV–visible transmittance were systematically evaluated. Modified nano-silica markedly improved pendulum hardness and scratch resistance, with hardness increasing by nearly 50%, while flexibility remained nearly unchanged. Although hydrophobicity and optical transmittance slightly decreased with increasing nano-silica content, the transmittance remained above 90% at 4 wt% loading. For KH-550 modified systems, strict pH control (pH 8.0) and ammonia removal were critical for sol stability. This work offers a feasible approach for fabricating scratch-resistant, transparent UV-curable coatings for optical plastics.

## 1. Introduction

Optical plastics are a class of polymeric materials that can be used as optical media [1]. The initial attempt to replace conventional optical glass with optical plastics for the fabrication of optical components dates back to the late 1930s and early 1940s in countries such as the United Kingdom and the United States, driven by the urgent demand for large-scale production of optical devices including gun sights, telescopes, and magnifiers during wartime [2,3,4]. In recent years, as optical plastics have been increasingly adopted worldwide, their inherent drawbacks have become more evident. Compared with optical glass, optical plastics generally exhibit lower surface hardness, making them susceptible to abrasion and scratching during service, which results in visible surface defects and limits their application in high performance optical components [5,6]. Therefore, improving the scratch and abrasion resistance of optical plastic surfaces has become a critical issue for expanding their application scope [7]. At present, one of the most effective approaches to enhance the surface durability of optical plastic components is surface modification, particularly through the deposition of transparent, scratch-resistant protective coatings [8,9,10]. Current research on transparent scratch-resistant coatings is mainly focused on polysiloxane-based systems and multifunctional acrylate-based hard coatings [11]. Meanwhile, polyurethane-based coatings have also attracted increasing attention for use in scratch-resistant transparent coatings on optical plastics due to their strong adhesion, tunable soft and hard segments, and the ability to tailor the mechanical and physical properties of the resulting films [12,13].

Ultraviolet (UV) curable coatings are a class of coatings that utilize ultraviolet radiation as the curing energy source [14,15]. Upon UV irradiation, the photo initiator undergoes decomposition to generate free radicals, which initiate polymerization reactions of the resin components, leading to rapid crosslinking and film formation. UV-curable coatings are typically composed of functionalized oligomers, reactive diluents, photo initiators, and various additives. Functionalized oligomers are photo-sensitive resins with relatively low molecular weights and contain reactive groups, such as carbon–carbon double bonds (C=C) or epoxy groups, which can participate in photopolymerization reactions [16,17,18,19]. These oligomers constitute the primary network structure of UV-curable coatings and play a decisive role in determining their physical and chemical properties [20]. Common types of photosensitive oligomers include pure acrylate resins, epoxy acrylates, polyether acrylates, polyester acrylates, unsaturated resins, polyurethane acrylates, silicone acrylates, and fluorinated acrylates [21,22,23].

Polyurethane acrylate (PUA) is a typical UV-curable oligomer synthesized through the reaction between isocyanate (–NCO) groups of polyisocyanates and hydroxyl groups of polyols, followed by the incorporation of photoreactive acrylate groups via hydroxyl-containing acrylates [24,25]. The molecular structure of PUA contains both urethane linkages and acrylate functional groups [26]. Consequently, UV-cured PUA coatings combine the excellent weather resistance and optical clarity of polyacrylates with the superior abrasion resistance, strong adhesion to plastic substrates, wide service temperature range, and good flexibility characteristic of polyurethanes [27,28]. Owing to these advantages, PUA-based coatings have attracted considerable attention and have been widely applied in plastic coatings, anticorrosion coatings, automotive topcoats, wood coatings, and architectural exterior coatings [29]. While optical plastics such as PMMA and PC are widely used, their application as high-performance protective layers is hindered by inherent brittleness (in PMMA) or poor solvent resistance (in PC). In this study, Polyurethane Acrylate (PUA) was selected as the matrix due to its unique segmented structure, which combines the toughness of carbamate groups with the optical clarity of polyacrylates. Unlike thermoplastic PMMA, the UV-curable PUA forms a dense cross-linked network that effectively anchors silane-modified nano-SiO_2_. This synergy allows for a significant enhancement in surface hardness and scratch resistance without compromising the flexibility and transparency, addressing the common trade-off issues found in traditional optical polymers.

Among various inorganic nanoparticles, nanoscale silicon dioxide (nano-SiO_2_) is one of the earliest inorganic nanomaterials to be systematically studied and practically applied [30]. China has become the fifth country, following the United States, the United Kingdom, Japan, and Germany, capable of large-scale industrial production of nano-SiO_2_. At present, relatively mature production technologies for nano-SiO_2_ have been established in China, and the material has been widely employed in the preparation of modified composite coatings [31,32]. Nano-SiO_2_ particles possess an extremely high specific surface area and surface energy, together with strong chemical reactivity, which enables the formation of stable three-dimensional network structures during the curing process of coatings [33,34]. As a result, the surface smoothness and mechanical strength of coatings can be significantly enhanced. In addition, the surfaces of nano-SiO_2_ particles are rich in silanol (Si–OH) groups and unsaturated residual bonds. These abundant Si–OH groups are capable of forming chemical bonds with functional groups in coating matrices, thereby markedly improving the thermal stability of the resulting coatings [35]. Moreover, nano-SiO_2_ exhibits unique optical properties, including strong infrared reflection and ultraviolet absorption capabilities [36]. Its reflectance for infrared radiation with wavelengths above 800 nm can exceed 70%, while its absorption of ultraviolet radiation below 400 nm can also reach over 70%. Incorporation of nano-SiO_2_ particles into coating systems can therefore effectively enhance thermal insulation performance and significantly improve resistance to ultraviolet induced aging and thermal aging, making nano-SiO_2_ an attractive functional additive for advanced protective and optical coatings [37,38,39].

In this work, we present a systematic and integrated approach to developing highly transparent, scratch-resistant coatings by bridging the gap between inorganic sol–gel synthesis and organic UV-curing technology. The novelty of this research lies in the precise kinetic control of TEOS hydrolysis and the subsequent comparative investigation of three distinct silane coupling agents (KH-550, KH-560, and KH-570) on the interfacial compatibility of nano-silica. Unlike conventional blending methods, our strategy leverages the synergistic effect of surface-functionalized silica and the PUA matrix to create a robust, covalently bonded hybrid network. By elucidating the underlying reinforcement mechanism, this study provides a scalable and efficient pathway to overcome the long-standing trade-off between optical clarity and mechanical durability in polymer optics.

## 2. Materials and Methods

### 2.1. Materials

Tetraethyl orthosilicate (TEOS), anhydrous ethanol, glacial acetic acid, ammonia solution, and ethyl acetate were purchased from Sinopharm Chemical Reagent Co., Ltd. (Shanghai, China). The silane coupling agent KH-550, KH560 and KH570 were purchased from Shanghai Yaohua Chemical Reagent Co., Ltd. (Shanghai, China), and PET plastic sheets were purchased from Shenzhen Jinkaixinrui Optoelectronics Co., Ltd. (Shenzhen, China).

### 2.2. Preparation and Modification of Silica Sol

First, tetraethyl orthosilicate (TEOS), deionized water, and anhydrous ethanol were weighed according to a molar ratio of 1:4:4 (0.1 mol TEOS, 0.4 mol deionized water, and 0.4 mol anhydrous ethanol). Half of the anhydrous ethanol was added to TEOS, while the other half was added to the deionized water. Subsequently, glacial acetic acid was added to the mixture of anhydrous ethanol and deionized water as an acidic catalyst, and the pH value was adjusted to 4.0. Finally, the solution was subjected to ultrasonic treatment at 50 kHz for 15 min to ensure thorough mixing.

After ultrasonic dispersion, the mixture containing glacial acetic acid, anhydrous ethanol, and deionized water was slowly added dropwise to the TEOS–ethanol mixture, which had also been ultrasonically dispersed. After 0.5 h, the silane coupling agent KH-550 was slowly introduced into the system at an amount corresponding to 25.0% of the TEOS content. The reaction system was then sealed to prevent solvent evaporation and stirred at 50 °C for 2 h. After completion of the reaction, a KH-550 modified silica sol was obtained and stored in a dry environment for further use.

The above procedure was repeated to prepare silica sol modified with the silane coupling agent KH-560 and KH570, which was also stored in a dry environment for subsequent use.

### 2.3. Preparation of Polyurethane Acrylate/Modified Nano-SiO_2_ Coatings

The PET plastic sheets used as substrates were cut into specimens with dimensions of 60 mm × 60 mm. The protective film on the surface was removed, and the substrate surfaces were cleaned by wiping with cotton swabs soaked in anhydrous ethanol to ensure surface cleanliness. The treated PET substrates were then placed on a flat laboratory bench for subsequent use.

The modified silica sol prepared via hydrolysis and condensation of tetraethyl orthosilicate (TEOS) as the inorganic precursor was mixed with the formulated polyurethane acrylate varnish at different mass ratios in small reagent bottles. The mixtures were then ultrasonically dispersed for 2 h. During this process, the bottles were kept strictly sealed, exposure to light was minimized, and moisture contamination was carefully avoided. After ultrasonic dispersion, the coating formulations were taken out and applied onto the prepared PET substrates using cotton swabs, followed by leveling for 2 min under horizontal conditions. After self-leveling on the PET substrate surface, the coated samples were cured under ultraviolet irradiation for 60 s in a UV-curing chamber, with an irradiation distance maintained at 45–55 cm, resulting in the formation of cured films on the PET substrates. After UV curing, the coated samples were carefully examined for surface defects such as unevenness or bubbles. Samples that met the quality requirements were labeled and prepared for subsequent performance testing.

### 2.4. Standardization of Silica Sol Concentration

In this study, 1 mol of tetraethyl orthosilicate (TEOS) theoretically requires 2 mol of water for complete hydrolysis. In contrast, the three silane coupling agents used require water at a molar ratio of 3:1 for hydrolysis. Therefore, according to the above formulation, the prepared silica sol still contains excess water. Since water is immiscible with ethyl acetate, the solvent in the polyurethane acrylate (PUA) varnish, phase separation between aqueous and organic phases occurs after mixing the silica sol with the polyurethane acrylate varnish. If the residual water in the system is not removed, the high surface tension of water during film curing can generate strong capillary forces, leading to particle agglomeration and consequently deteriorating the optical properties (such as transmittance) of the resulting coating. Therefore, it is necessary to remove the excess water from the system during the experiment. The dehydration process is based on the azeotropic distillation principle of the water–ethanol–ethyl acetate system. During UV curing, the heat released by the ultraviolet curing instrument is utilized to remove water from the system. The azeotropic boiling point of the water–ethanol–ethyl acetate system is 70.3 °C, and the mass fractions of water, ethanol, and ethyl acetate in the azeotrope are 7.8%, 9.0%, and 83.2%, respectively.

### 2.5. Characterization Techniques

#### 2.5.1. FT-IR Characterization of Modified Silica Sol

In this test, the potassium bromide (KBr) pellet method was employed. The modified silica sol sample was first coated onto a prepressed KBr pellet, and its infrared absorption spectrum was then recorded using a Fourier transform infrared (FT-IR) spectrometer (Nicolet 6700, Madison, WI, USA). The obtained spectra were subsequently analyzed.

#### 2.5.2. Particle Size Distribution of Nano-SiO_2_ Particles

The particle size distribution of nano-SiO_2_ particles was measured using a Malvern particle size analyzer (Zetasizer Nano, Malvern, UK). A small amount of unmodified and modified silica sol was separately dropped into quartz cuvettes, and the particle size distribution of the nano-SiO_2_ particles in the silica sol was measured at 25 °C.

#### 2.5.3. TEM Characterization of Modified Silica Sol

Samples of unmodified silica sol and silica sol modified with different silane coupling agents were appropriately diluted with anhydrous ethanol and then dropped onto copper grids. After natural drying, the dispersion state of the nano-SiO_2_ particles was observed using a field emission transmission electron microscope (TEM, JEM-2100F, Tokyo, Japan).

#### 2.5.4. SEM Characterization of Composite Coatings

Coated samples with two different composite coatings were cut into specimens with dimensions of 5 mm × 5 mm. After gold sputtering, the surface morphology of the composite coatings was observed using a field emission scanning electron microscope (SEM, Zeiss Ultra Plus, Oberkochen, Germany).

#### 2.5.5. Contact Angle Measurement of Composite Coatings

The contact angle measurements were carried out using a static contact angle goniometer (JC2000C, Shanghai, China). At room temperature, the surface contact angles of the composite coatings containing silane-coupling-agent-modified silica sol were measured using deionized water and oil (diiodomethane, CH_2_I_2_), and the surface energy was subsequently calculated. The UV-cured coating films were placed on the sample stage of the contact angle goniometer. The software was activated, and the light intensity and focal length were adjusted to obtain a clear droplet profile. The static drop method was employed to measure the contact angle with water first, followed by measurements using CH_2_I_2_ under the same conditions. Each contact angle measurement for water and CH_2_I_2_ was repeated three times, and the average values were used for calculations. The surface energy of the composite coatings was calculated based on the measured contact angles of water and CH_2_I_2_. The surface energy was calculated using the following equations:

$$\left\{\begin{array}{l} \gamma_{L_{1}}\left(1+cos\;\theta_{H_{2}O}\right)=2(\sqrt{\gamma_{L_{1}}^{d}\times \gamma_{S}^{d}}+\sqrt{\gamma_{L_{1}}^{p}\times \gamma_{S}^{p}})\\ \gamma_{L_{2}}\left(1+cos\;\theta_{CH_{2}I_{2}}\right)=2(\sqrt{\gamma_{L_{2}}^{d}\times \gamma_{S}^{d}}+\sqrt{\gamma_{L_{2}}^{p}\times \gamma_{S}^{p}})\\ \gamma_{S}=\gamma_{S}^{d}+\gamma_{S}^{p} \end{array}\right.$$

where

$\gamma_{L_{1}}$—surface tension of water (mN/m);

$\gamma_{L_{2}}$—surface tension of CH_2_I_2_ (mN/m);

$cos\;\theta_{H_{2}O}$—contact angle of the coating with water (°);

$cos\;\theta_{CH_{2}I_{2}}$—contact angle of the coating with CH_2_I_2_ (°);

$\gamma_{L_{1}}^{d}$—dispersive component of the surface tension of water (mN/m);

$\gamma_{L_{1}}^{p}$—polar component of the surface tension of water (mN/m);

$\gamma_{L_{2}}^{d}$—dispersive component of the surface tension of CH_2_I_2_ (mN/m);

$\gamma_{L_{2}}^{p}$—polar component of the surface tension of CH_2_I_2_ (mN/m);

$\gamma_{S}$—surface free energy of the coating (mN/m);

$\gamma_{S}^{d}$—dispersive component of the surface free energy of the coating (mN/m);

$\gamma_{S}^{p}$—polar component of the surface free energy of the coating (mN/m).

#### 2.5.6. Hardness Test of Composite Coatings

The hardness of the composite coatings was evaluated according to the Chinese national standard Paint films—Determination of film hardness—Pendulum damping test (GB/T 1730-93) [40] using a dual pendulum damping method. A QBY-type pendulum hardness tester was employed for the measurements.

Prior to testing, the two pendulums of the hardness tester were calibrated using an uncoated blank substrate. The damping time required for the pendulum swing amplitude to decrease from 5° to 2° on the uncoated substrate was adjusted to (440 ± 6) s. If the measured value was outside this range, the positions of the two counterweights on the front and rear pendulums were simultaneously adjusted until the calibration requirement was satisfied. Subsequently, the supporting steel balls were carefully cleaned using a soft silk cloth or cotton paper soaked in diethyl ether.

For hardness measurements, the coated sample was placed horizontally on the test platform with the coating surface facing upward. The pendulum was gently lowered onto the coating surface, and the alignment between the scale zero point and the pendulum tip at rest was checked and adjusted if necessary. After positioning, the pendulum was deflected to 5.5° and released. Timing was initiated when the pendulum passed 5° and stopped at 2°. The time required for the swing amplitude to decay from 5° to 2° was recorded. The coating hardness was calculated according to the following equation:

$$X=t/t_{0}$$

where

X—hardness of the tested coating;

t—damping time for the pendulum swing from 5° to 2° on the coated substrate (s);

t_0_—damping time for the pendulum swing from 5° to 2° on the uncoated substrate (s).

#### 2.5.7. Adhesion Test of Composite Coatings

The adhesion of the composite coatings was evaluated according to the Chinese national standard Paints and varnishes—Cross-cut test (GB/T 9286-1998) [41]. Prior to testing, the cutting blade was inspected and sharpened or replaced when necessary to ensure proper cutting performance.

During the test, the coated sample was placed on a rigid and flat horizontal workbench to prevent deformation. The cutting tool was held perpendicular to the coating surface, and uniform force was applied to produce a specified number of parallel cuts at an even spacing and cutting speed. All cuts were required to penetrate through the coating to the substrate surface. The procedure was then repeated with the same number of cuts made perpendicular to the first set, forming a grid pattern.

A soft brush was used to gently sweep along each diagonal of the grid several times in both backward and forward directions. A piece of adhesive tape approximately 75 mm in length was then prepared. The centre of the tape was placed over the grid area with the tape aligned parallel to one set of cuts. The tape was pressed firmly over the grid region, ensuring that its length extended at least 20 mm beyond the grid. Within 5 min of tape application, the free end of the tape was pulled off smoothly at an angle as close as possible to 60° within 0.5–1.0 s.

The cut area was examined under good lighting conditions using a 4× magnifying glass. The adhesion of the coating to the PET substrate was evaluated based on the degree of coating detachment in the grid area. Adhesion was classified into six grades (0–5), and the grading criteria are listed in Table 1.

#### 2.5.8. Transmittance Measurement of Composite Coatings

The optical transmittance of the composite coatings was measured using a UV–Vis spectrophotometer (UV-2550, Shimadzu, Kyoto, Japan). The Shimadzu UV-2550 spectrophotometer, combined with the UV Probe 2.7 operating software and an advanced ultra-low stray light system, provides high performance and convenient operation. During the measurements, PET substrates coated with the modified coatings were cut into specimens with dimensions of 4 cm × 1 cm to fit the cuvette size. The samples were then placed in the sample holder, and the scanning wavelength range was set from 300 to 900 nm to determine the ultraviolet–visible transmittance of the composite coatings.

#### 2.5.9. Scratch Resistance Test of Composite Coatings

**Figure 1:**
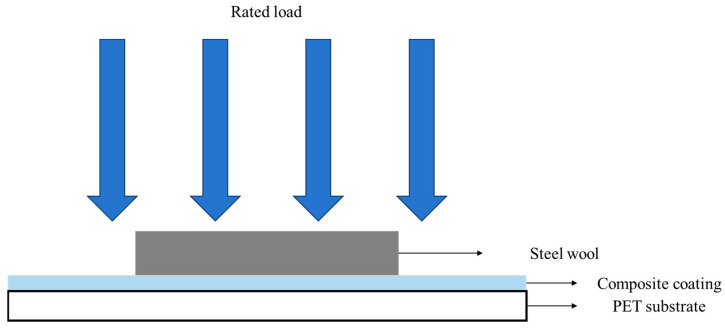
Diagram of Homemade Scratch Resistance Testing Device.

The scratch resistance of the composite coatings was evaluated using a self-designed apparatus for scratch resistance testing, as schematically illustrated in Figure 1 Under a selected normal load (150 g/cm^2^ in this study), steel wool was used to rub the surface of the composite coatings at a constant frequency. The number of rubbing cycles required to produce visible scratches on the coating surface was recorded.

Subsequently, the integral area of the UV–Vis transmittance curves of the composite coatings in the wavelength range of 300–900 nm was calculated for different rubbing cycles, and the optical loss rate of the composite coatings was determined. The calculation formula is given as follows:

$$d=\frac{S_{0}-S_{n}}{S_{0}}\times 100\%$$

where

*S_0_*—integral area of the transmittance curve of the coating without steel wool abrasion;

*Sₙ*—integral area of the transmittance curve of the coating after steel wool abrasion;

*n*—number of rubbing cycles.

Finally, the surface morphologies of the composite coatings with different nano-SiO_2_ contents after the same number of rubbing cycles were observed using a polarized optical microscope.

## 3. Results

### 3.1. Reaction Mechanism

**Figure 2:**
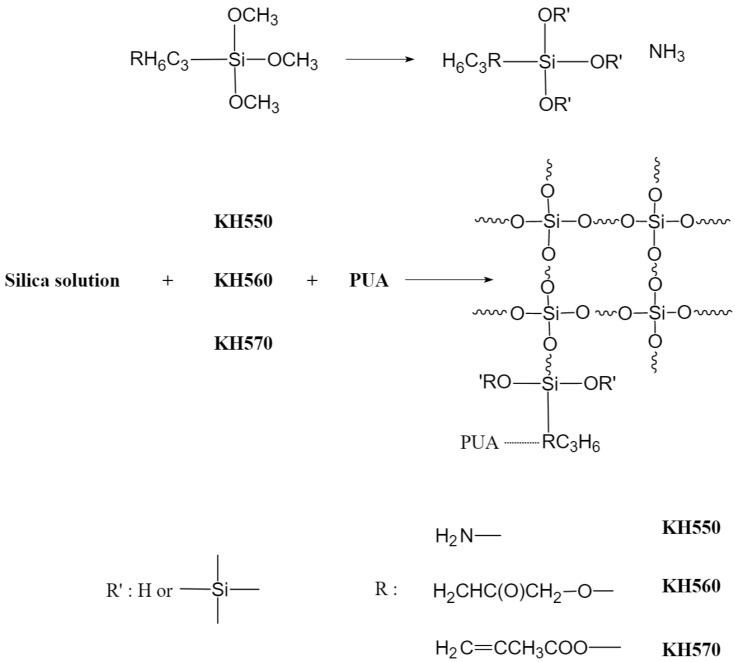
Copolymerization mechanism with TEOS and PUA.

The hydrolysis and condensation reactions of TEOS in an alkaline environment can be interpreted based on a nucleophilic substitution mechanism. Figure 2 schematically presents the co-polymerization mechanism between TEOS, various silane coupling agents, and PUA.

### 3.2. Effect of pH on Silica Sol Stability

When the hydrolysis and condensation reactions of TEOS are carried out under alkaline conditions, the hydrolysis rate is much lower than the condensation rate. The condensation reaction mainly occurs between silanol groups (Si–OH) and silicate ester groups, leading to the formation of a network-structured colloidal polymer. During the hydrolysis–condensation process of TEOS, strict control of the pH value of the reaction system is crucial. A slight excess of the alkaline catalyst can significantly affect the reaction, resulting in the formation of dense colloidal particles in the solution or even direct gelation. Therefore, it is necessary to determine an appropriate pH range for the reaction system. In this study, a series of parallel experiments was conducted by adding different amounts of the alkaline catalyst ammonium hydroxide to the system. The transparency of the resulting silica sols under different pH conditions was compared to identify the optimal pH value for the hydrolysis and condensation of TEOS under alkaline conditions.

**Figure 3:**
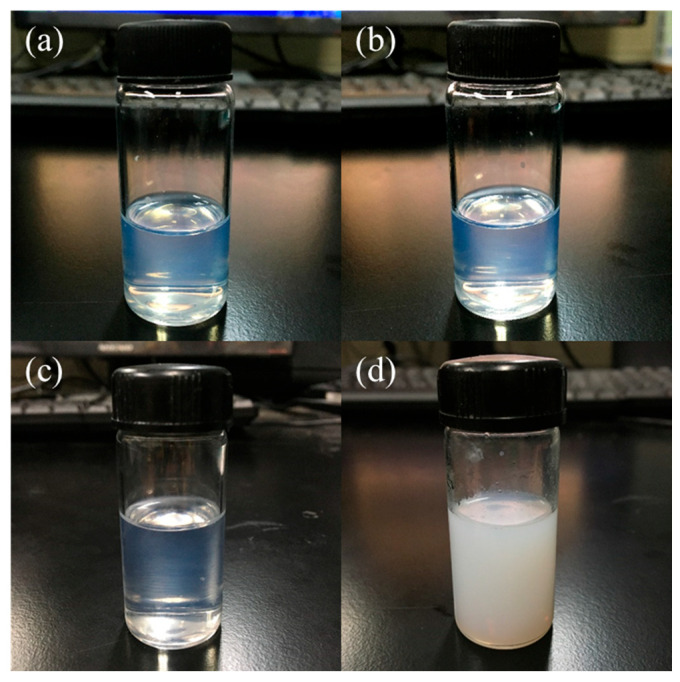
Unmodified silica sol (**a**) after removal of ammonia; (**b**) after removal of ammonia and storage at room temperature for 30 days (**c**) without removal of ammonia (**d**) without removal of ammonia after standing for 2 h.

As shown in Table 2, when the pH value of the reaction system was controlled at 8.0, the silica sol prepared by the Stöber method under alkaline conditions appeared as a light blue transparent liquid, and no obvious particulate matter was observed in the sol. When the amount of aqueous ammonia was increased and the pH value of the reaction system reached 9.0, white flocculent precipitates began to form during the reaction process. When the pH value was further increased to 10.0, the white flocculent precipitates in the solution became more pronounced. At a pH value of 11.0, a jelly-like milky white gel was directly formed. Therefore, the appropriate pH value for the reaction system was determined to be 8.0. In addition, after the completion of TEOS hydrolysis for silica sol preparation under alkaline conditions, it is necessary to remove the excess ammonia from the reaction system so that the prepared silica sol is maintained in a near neutral environment. A small amount of unmodified silica sol with and without ammonia removal was transferred into transparent vials and stored at room temperature. It was observed that the silica sol without ammonia removal gradually underwent gelation over time: the light blue transparent liquid progressively became turbid and milky white, and almost completely gelled after 2 h (Figure 3d). In contrast, the silica sol from which excess ammonia had been removed remained clear even after storage at room temperature for 30 days, with only a slight deepening of color (Figure 3b). When TEOS undergoes hydrolysis and condensation reactions in a near neutral environment, both the hydrolysis rate and the condensation rate are at their lowest levels compared with nonacidic conditions. The presence of excess ammonia keeps the sol in an alkaline environment, thereby promoting further condensation and even aggregation between reaction products, as well as between products and reactants, ultimately leading to gelation and loss of usability. The significant difference between hydrolysis and condensation rates is governed by the pH of the medium. Under the alkaline conditions (pH 8.0) employed in this study, the nucleophilic attack of hydroxyl ions (OH^−^) on the silicon atom promotes a rapid condensation process that exceeds the rate of hydrolysis. This kinetic profile favors the formation of discrete, dense spherical particles rather than an extended linear network, which is consistent with the observed light-blue transparent sol. Therefore, incorporating a step to remove excess ammonia from the reaction system is particularly critical in the preparation of silica sol. The precise control of ammonium hydroxide dosage is critical. An excess of NH_3_·H_2_O (leading to pH > 9.0) would shift the kinetic balance drastically toward condensation. In such a high-alkalinity environment, the rapid formation of Si-O-Si networks and the simultaneous compression of the electrical double layer would lead to uncontrolled particle aggregation. Physically, this results in the loss of the sol’s characteristic transparency and the formation of white flocculent precipitates or a rigid gel, which is unsuitable for the subsequent preparation of high-quality optical coatings.

### 3.3. FT-IR Analysis of Modified Silica Sol

**Figure 4:**
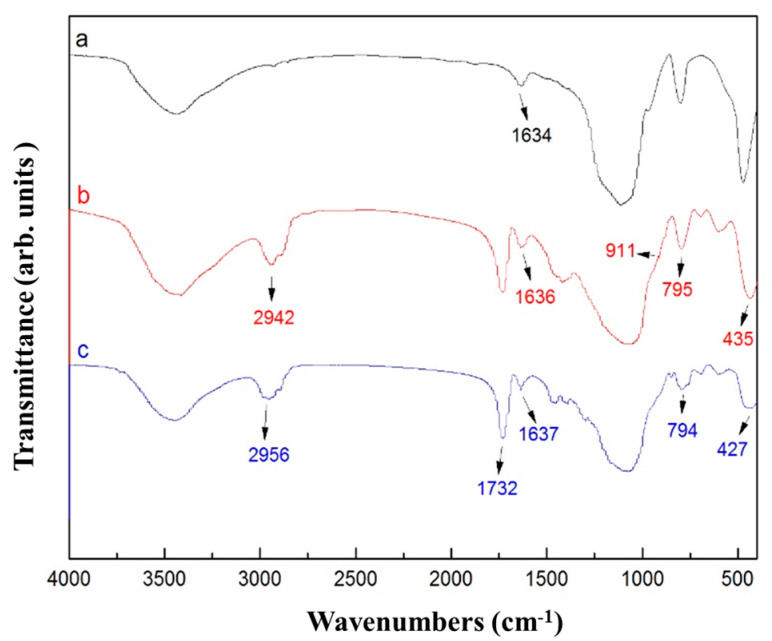
FT-IR spectra of the unmodified silica sol (a) and silica sols modified with KH-560 (b) and KH-570 (c).

Figure 4 shows the FT-IR spectra of the unmodified silica sol and the silica sols modified with KH-560 and KH-570. Curve a corresponds to the unmodified silica sol, while curves b and c represent the silica sols modified with KH-560 and KH-570, respectively. In curve a of Figure 4, the absorption band observed at 1634 cm^−1^ is attributed to the bending vibration of adsorbed water on the surface of the nano-silica particles. After modification with KH-560 and KH-570, this band slightly shifts to 1636 cm^−1^ and 1637 cm^−1^, respectively. This shift can be assigned to the change in the surface chemical environment and hydrogen bonding interactions as silane coupling agents react with the surface silanol groups, thereby altering the adsorption state of water molecules. As observed from the spectra, compared with curve a, new absorption peaks appear in curves b and c at 2942 cm^−1^ and 2956 cm^−1^, respectively, which can be attributed to the vibrational absorption of C–H bonds. In addition, the peaks at 795 cm^−1^ and 435 cm^−1^ in curve b, as well as those at 794 cm^−1^ and 427 cm^−1^ in curve c, correspond to the bending and rocking vibrational modes of Si–O bonds. Compared with the FT-IR spectrum of the unmodified silica sol, a new absorption peak appears at 911 cm^−1^ in curve b and at 1732 cm^−1^ in curve c, which are assigned to the asymmetric stretching vibration of the epoxy ring and the stretching vibration of the C=O double bond, respectively. Although the absolute concentration of surface silanol groups was not directly titrated, the relative decrease in the intensity of the Si-OH stretching vibration (around 960 cm^−1^) after modification with KH-560 and KH-570 provides qualitative evidence of the partial consumption of silanol groups during the grafting process. These results indicate that the silane coupling agents were successfully grafted onto the surface of nano-SiO_2_ particles.

### 3.4. Particle Size Distribution of Silica Sol

**Figure 5:**
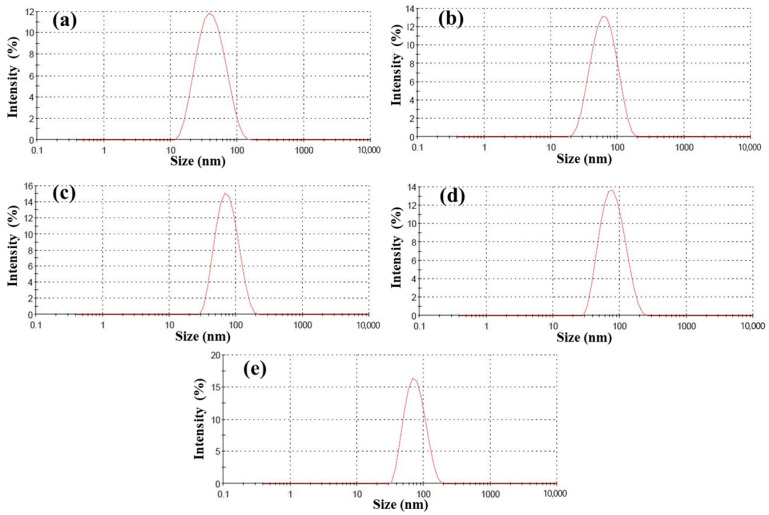
Particle size distribution of nano-SiO_2_ particles in silica sol under acidic (**a**) and alkaline (**b**) conditions; silica sols modified with KH550 (**c**), KH-560 (**d**) and KH-570 (**e**).

Figure 5 shows the particle size distribution of silica sol. For the unmodified silica sol prepared under alkaline conditions, the average particle size of the nano-SiO_2_ particles was 56.5 nm, with a polydispersity index (PDI) of 0.152. In contrast, the unmodified silica sol prepared under acidic conditions exhibited an average particle size of 36.2 nm and a PDI value of 0.188. After modification with KH-550, the average particle size in the nano-silica sol increased to 68.7 nm, while the PDI decreased to 0.118. Similarly, after modification with KH-560 and KH-570, the average particle sizes increased to 71.2 nm and 69.5 nm, with corresponding PDI values of 0.140 and 0.104, respectively. These results indicate that the average particle size of the nano-SiO_2_ particles slightly increased after modification with silane coupling agents. Meanwhile, both the modified and unmodified silica sols exhibited relatively narrow particle size distributions, suggesting good dispersion of the nanoparticles in the dispersion medium.

### 3.5. TEM and SEM Morphologies of Silica Sol

**Figure 6:**
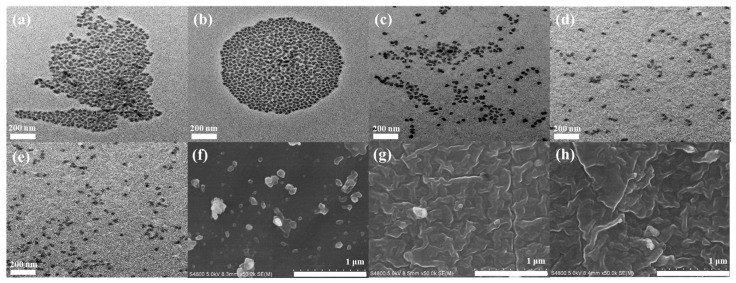
TEM images of unmodified silica sol prepared under acidic (**a**) and alkaline (**b**) conditions; silica sols modified with KH550 (**c**), KH-560 (**d**) and KH-570 (**e**); SEM images of composite coatings modified with KH-550 (**f**), KH-560 (**g**), and KH-570 (**h**).

As shown in Figure 6, severe aggregation of nano-SiO_2_ particles is observed in the unmodified silica sol prepared under both acidic and alkaline conditions. In contrast, the nano-SiO_2_ particles in the silica sols modified with silane coupling agents exhibit a much more uniform dispersion, with significantly reduced particle aggregation and predominantly nanoscale dispersion. However, slight particle agglomeration can still be observed in the composite coating modified with KH-550, which may be attributed to the relatively low dosage of KH-550 used in the modification process.

### 3.6. Water Contact Angle and Surface Energy of Composite Coatings

**Figure 7:**
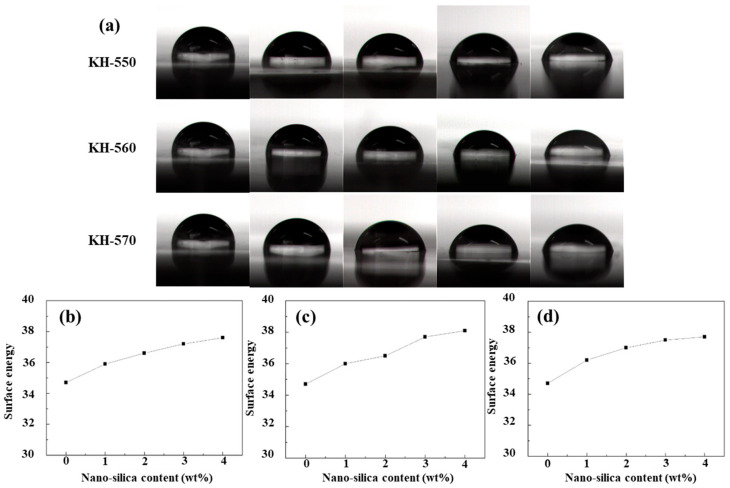
Contact angle images of composite coatings containing different contents of silane-coupling-agent-modified nano-SiO_2_ (**a**), from left to right: 0 wt%, 2 wt%, 3 wt%, and 4 wt%. Surface energy of composite coatings with different loadings of KH-550 (**b**), KH-560 (**c**), and KH-570 (**d**) modified nano-SiO_2_.

As shown in Figure 7a, the water contact angle of the blank polyurethane acrylate (PUA) coating is relatively high, reaching 88.0°. After introducing silane-coupling-agent-modified nano-SiO_2_ into the coating system, the water contact angle of the coatings gradually decreases. When the contents of modified nano-SiO_2_ are 1 wt%, 2 wt%, 3 wt%, and 4 wt%, the water contact angles of the coatings containing KH-550-modified nano-SiO_2_ are 84.5°, 82.5°, 78.0°, and 75.5°, respectively. For coatings containing KH-560-modified nano-SiO_2_, the corresponding contact angles are 85.5°, 83.0°, 78.5°, and 76.0°, while those for coatings containing KH-570-modified nano-SiO_2_ are 85.0°, 82.0°, 78.5°, and 75.5°, respectively. These results indicate a gradual reduction in the hydrophobicity of the composite coatings.

Furthermore, as shown in Figure 7b–d and Table 3, the surface energy of the composite coatings increases to some extent with increasing content of modified nano-SiO_2_, although the increasing trend is not pronounced. After incorporating modified nano-SiO_2_ into the coating system and curing under UV irradiation, the water contact angle of the coatings decreases progressively, accompanied by a weakening of hydrophobicity. This behavior may be attributed to the enrichment of partially unmodified nano-SiO_2_ particles at the coating surface. These unmodified nanoparticles retain a large number of surfaces silanol (Si–OH) groups, which impart hydrophilic characteristics. While the precise density of Si–OH groups was not measured in this study, it is well-established in the literature that the surface of Stober-derived silica typically contains 4–5 OH/nm^2^ [42]. The observed decrease in water contact angle suggests that despite the modification, a sufficient density of residual hydroxyl groups remains to influence the surface energy. In addition, surface tension effects within the system can drive particles with lower surface energy to migrate toward the outer layer of the coating. Consequently, the hydrophobicity of the composite coatings gradually decreases, while their surface energy exhibits a corresponding increasing trend.

### 3.7. Hardness of Composite Coatings

**Figure 8:**
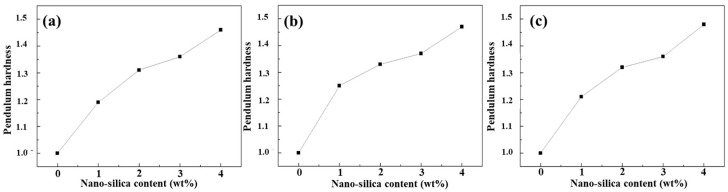
Hardness of composite coating pendulums containing modified nano-silica with different load quantities KH-550 (**a**), KH-560 (**b**), and KH-570 (**c**).

By combining the pendulum hardness values shown in Figure 8 and Table 4, it can be observed that the hardness of the composite coatings exhibits a pronounced increasing trend with increasing content of modified nano-SiO_2_. When the loading of modified nano-SiO_2_ reaches 4 wt%, the pendulum hardness of the composite coatings increases by nearly 50%. Moreover, no significant difference in pendulum hardness is observed between coatings containing nano-SiO_2_ modified with different silane coupling agents. This enhancement can be attributed to the intrinsic rigidity of nano-SiO_2_ particles. When introduced into the coating system, these rigid nanoparticles effectively improve the stiffness of the cured coatings. In addition, surface-modified nano-SiO_2_ particles are more uniformly dispersed within the coating matrix and may form chemical linkages with the organic matrix through silane coupling agents or physical crosslinking via hydrogen bonding. As a result, the mechanical strength of the organic matrix in the UV-curable coating is enhanced, leading to a corresponding increase in the pendulum hardness of the cured coatings.

### 3.8. Optical Transmittance of Composite Coatings

**Figure 9:**
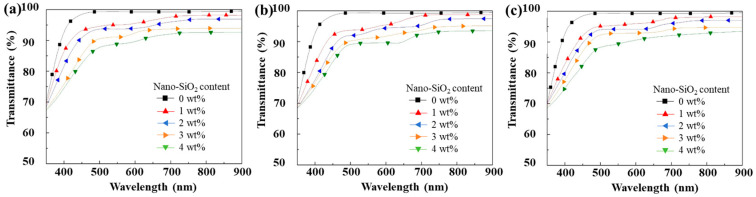
Optical Transmittance of composite coating pendulums containing modified nano-silica with different load quantities KH-550 (**a**), KH-560 (**b**), and KH-570 (**c**).

Figure 9 shows the UV–Vis transmittance spectra of the composite coatings. After incorporating modified nano-SiO_2_ into the coating formulation, the resulting hybrid coatings remain homogeneous and transparent, even when the content of modified nano-SiO_2_ reaches 4 wt%. As can be observed from the spectra, the optical transmittance of the coatings in the visible region gradually decreases with increasing content of modified nano-SiO_2_. Specifically, for composite coatings containing KH-550-modified nano-SiO_2_, the transmittance at a wavelength of 800 nm decreases from 98.14% for the blank PUA coating to 92.52%. For coatings containing KH-560-modified nano-SiO_2_, the transmittance at 800 nm decreases from 99.30% to 93.40%, while for coatings containing KH-570-modified nano-SiO_2_, the corresponding value decreases from 99.30% to 93.44%. Nevertheless, the transmittance of all composite coatings remains above 90%, indicating that a relatively high level of optical transparency is maintained. This decrease in transmittance can be attributed to the fact that complete nanoscale dispersion of nano-SiO_2_ particles in the coating system cannot be fully guaranteed, and that the grafting efficiency of silane coupling agents on the nano-SiO_2_ surface is not 100%. As a result, a considerable number of silanol (Si–OH) groups remain on the particle surfaces, which can undergo partial condensation with each other to form micron-scale aggregates. These aggregates scatter incident light, leading to a reduction in the UV–Vis transmittance of the composite coatings. Furthermore, as the amount of modified SiO_2_ introduced in the form of silica sol increases, light scattering is further enhanced, resulting in a continued decrease in optical transmittance.

### 3.9. Scratch Resistance of Composite Coatings

**Figure 10:**
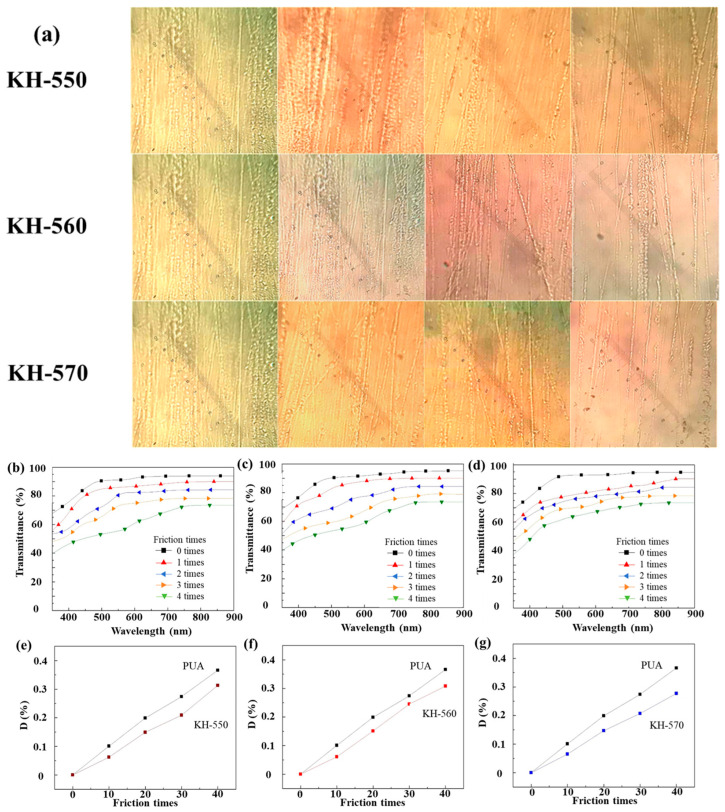
(**a**) Surface morphologies of composite coatings containing different contents of silane-coupling-agent-modified nano-SiO_2_ after the same number of rubbing cycles (from left to right: 0 wt%, 2 wt%, 3 wt%, and 4 wt%); UV–Vis transmittance of composite coatings with different loadings of KH-550 (**b**,**e**), KH-560 (**c**,**f**), KH570 (**d**,**g**).

Figure 10 presents polarized optical microscope images of the surface morphologies of the composite coatings after 40 rubbing cycles with steel wool under a constant load of 250 g/cm^2^. As can be clearly observed from the images, the number and depth of scratches on the coating surfaces decrease with increasing content of modified nano-SiO_2_. This improvement can be attributed to the incorporation of inorganic nanoparticles with high rigidity and high mechanical strength into the coating system, which effectively enhances the scratch resistance of the coatings. The significant improvement in scratch resistance stems from a dual reinforcement mechanism: First, the inorganic rigid skeleton formed by nano-silica particles effectively bears vertical loads, preventing deep penetration by the indenter. Second, silicate covalent integration achieved through silane coupling agent modification creates a robust particle-polyurethane matrix interface during scratching. This ensures efficient energy dissipation while deflecting crack tips, thereby preventing catastrophic damage. Silicate covalent integration achieved through silane coupling agent modification creates a tough interface between particles and the PUA matrix. This ensures efficient energy dissipation during scratching while forcing crack tip deflection, thereby preventing catastrophic material delamination. Additionally, increased crosslink density within the hybrid network significantly reduces plastic flow of polymer chains under shear stress.

The data listed in Table 5, under a rated load of 150 g/cm^2^ using steel wool for abrasion testing, the surface of the blank PUA cured coating without modified nano-SiO_2_ is highly susceptible to the formation of obvious scratches after abrasion. With increasing content of modified nano-SiO_2_, the number of rubbing cycles required to produce visible scratches on the surface of the composite coatings increases progressively. When the loading of modified nano-SiO_2_ reaches 4 wt%, steel wool can no longer easily produce clear and distinct scratches on the coating surface. These results demonstrate that the incorporation of surface-modified nano-SiO_2_ particles into the solvent-based PUA coating system effectively enhances the scratch resistance of the coatings. This improvement can be attributed to the increasing content of the inorganic silica phase with high hardness as the amount of modified nano-SiO_2_ increases. In addition, the presence of silane coupling agents promotes interfacial crosslinking between the organic and inorganic phases, resulting in a denser coating structure and thereby significantly improving the scratch resistance of the composite coatings.

As can be clearly observed from Figure 10b–d, for the same coating, the optical transmittance gradually decreases with increasing number of rubbing cycles. However, at the same number of rubbing cycles, the optical loss rate of the composite coatings containing modified nano-SiO_2_ is significantly lower than that of the blank PUA coating. After 40 rubbing cycles with steel wool, the optical loss rate of the blank PUA coating reaches 36.6%, whereas the optical loss rates of the composite coatings containing 3 wt% of KH-550-, KH-560-, and KH-570-modified nano-SiO_2_ are reduced to 31.3%, 30.8%, and 27.7%, respectively (Figure 10e–g). From a quantitative perspective, this enhancement is evidenced by a substantial reduction in scratch depth and width (Figure 10a). The rigid SiO_2_ particles serve as load-bearing elements that increase the critical load of the surface, thereby restricting the plastic flow of the polymer chains. This synergy between the inorganic phase and the UV-cured matrix ensures the long-term durability of the optical plastics in demanding environments. These results demonstrate that the incorporation of inorganic nano-SiO_2_ with high hardness and high rigidity into the organic matrix significantly enhances the scratch resistance of the coatings.

## 4. Conclusions

In this work, nano-SiO_2_ particles in silica sol were graft-modified using three different silane coupling agents and subsequently incorporated into a UV-curable polyurethane acrylate (PUA) coating system. On the premise of maintaining high optical transparency, composite coatings with enhanced scratch resistance were successfully prepared. Through the modification of nano-SiO_2_ sols and the performance evaluation of the resulting composite coatings, it was demonstrated that grafting silane coupling agents onto the surface of nano-SiO_2_ particles effectively improves their dispersion in the organic phase and significantly reduces particle agglomeration. Moreover, graft modification introduces active functional groups such as C=C unsaturated double bonds, epoxy groups, and amine groups onto the nano-SiO_2_ surface, thereby enhancing the interfacial reactivity between the nanoparticles and the PUA matrix.

When silica sol was prepared via TEOS hydrolysis under acidic conditions, the pH value of the system should be controlled at 4.0, which ensures a relatively fast hydrolysis rate of TEOS while maintaining a slow condensation rate of the hydrolysis products. In addition, the storage stability of the silica sol modified with KH-560 was superior to that of the silica sol modified with KH-570.

For silica sol prepared by TEOS hydrolysis under alkaline conditions, the pH value of the reaction system should be controlled at 8.0 to prevent gelation during the reaction process. During silica sol modification, a KH-550 dosage of 2.0 wt% relative to TEOS was found to be appropriate, as it avoids gelation within a short storage period. Furthermore, removing excess ammonia from the system to maintain the silica sol in a near-neutral environment can significantly improve its storage stability.

Incorporation of the modified nano-SiO_2_ into the UV-curable PUA coating system enhances the pendulum hardness, surface energy, and scratch resistance of the resulting coatings. Increasing the content of modified nano-SiO_2_ further improves these mechanical and surface properties. Although the optical transmittance of the composite coatings decreases to some extent with increasing nano-SiO_2_ content, the transmittance remains above 90% even at a nano-SiO_2_ loading of 4 wt%. In addition, no significant change in coating flexibility was observed. After 40 rubbing cycles under a load of 250 g/cm^2^, the optical loss rates of the composite coatings containing 3 wt% of the three modified nano-SiO_2_ types were 30.8%, 27.8%, and 31.3%, respectively, which are 5.8, 8.9, and 5.3 percentage points lower than that of the blank PUA coating under identical conditions.

Overall, this study demonstrates the successful development of a UV-curable coating that can be applied to substrate surfaces to improve scratch resistance, providing a novel strategy for enhancing the scratch resistance of optical plastics.

## Figures and Tables

**Table 1:** Classification and Description of Cross-Cut Adhesion Test Results.

Grade	Description
0	The edges of the cuts are completely smooth, and none of the squares is detached.
1	Slight detachment of the coating occurs at the intersections of the cuts; the affected area of a single cross-cut does not exceed 5%.
2	Detachment of the coating occurs at the intersections of the cuts and/or along the cut edges; the affected cross-cut area is greater than 5% but does not exceed 15%.
3	The coating partially or completely detaches from the cut edges in large flakes and/or partially or completely peels off at different locations; the affected cross-cut area is greater than 15% but does not exceed 35%.
4	Large flakes of the coating detach along the cut edges and/or partially or completely detach from some squares; the affected cross-cut area is greater than 35% but does not exceed 65%.
5	The degree of coating detachment exceeds Grade 4.

**Table 2:** Effect of pH on Silica Sol Stability.

pH	Transparency of the Sol
8.0	Light blue, transparent
9.0	White flocculent precipitate
10.0	Distinct white flocculent precipitate
11.0	Milky white gel

**Table 3:** Calculated surface energy values of composite coatings with different loadings of silane-coupling-agent-modified nano-SiO_2_.

	PUA	PUA + 1 wt% SiO_2_	PUA + 2 wt% SiO_2_	PUA + 3 wt% SiO_2_	PUA + 4 wt% SiO_2_
KH550	34.7	35.9	36.6	37.2	37.6
KH560	34.7	36.0	36.5	37.7	38.1
KH570	34.7	36.2	37.0	37.5	37.7

**Table 4:** Pendulum hardness values of composite coatings with different loadings of silane-coupling-agent-modified nano-SiO_2_.

	PUA	PUA + 1 wt% SiO_2_	PUA + 2 wt% SiO_2_	PUA + 3 wt% SiO_2_	PUA + 4 wt% SiO_2_
KH550	1.00	1.19	1.31	1.36	1.46
KH560	1.00	1.25	1.33	1.37	1.47
KH570	1.00	1.21	1.32	1.36	1.48

**Table 5:** Number of rubbing cycles required to produce visible scratches on composite coatings with different loadings of silane-coupling-agent-modified nano-SiO_2_.

	PUA	PUA + 1 wt% SiO_2_	PUA + 2 wt% SiO_2_	PUA + 3 wt% SiO_2_	PUA + 4 wt% SiO_2_
KH550	2	10	20	35	67
KH560	2	11	20	33	68
KH570	2	10	21	35	69

## Data Availability

The raw data supporting the conclusions of this article will be made available by the authors on request.
